# Prasugrel, a new medicine for preventing blockages in the arteries

**DOI:** 10.1107/S1600536810017095

**Published:** 2010-05-15

**Authors:** Zhi-Mei Wang, Jian Zhao, Gang Xu

**Affiliations:** a School of Chemistry and Chemical Engineering, Southeast University, Nanjing 211189, People’s Republic of China

## Abstract

Prasugrel {systematic name: 5-[(2-cyclo­propyl­carbon­yl)(2-fluoro­phen­yl)meth­yl]-4,5,6,7-tetra­hydro­thieno[3,2-*c*]pyridin-2-yl acetate}, C_20_H_20_FNO_3_S, is a new third-generation thienopyridine which was recently approved for clinical use as a more potent blocker of the platelet P2Y_12_ receptor than clopidogrel, which was previously used for this purpose. The mol­ecule features a tetra­hydro­thienopyridine system with the tetra­hydro­pyridine ring showing a half-chair conformation; the dihedral angle formed by the the planes of the benzene and thio­phene rings is 83.17 (3)°.

## Related literature

For the biological activity of the title compound, see: Farid *et al.* (2008 ▶). For details of the synthesis, see: Sun *et al.* (2009 ▶).
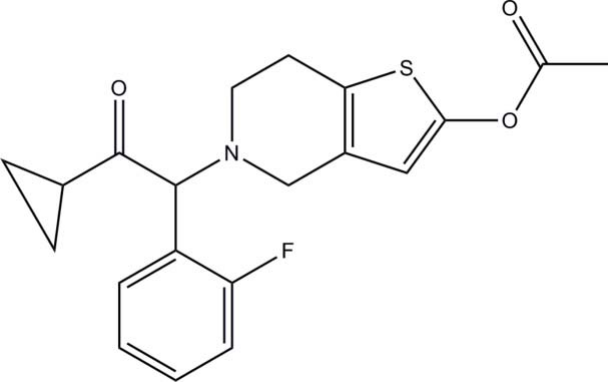


## Experimental

### 

#### Crystal data

C_20_H_20_FNO_3_S
*M* *_r_* = 373.43
Triclinic, 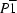

*a* = 7.910 (2) Å
*b* = 9.943 (3) Å
*c* = 12.450 (4) Å
α = 112.938 (5)°
β = 90.644 (5)°
γ = 92.591 (6)°
*V* = 900.3 (5) Å^3^
*Z* = 2
Mo *K*α radiation
μ = 0.21 mm^−1^
*T* = 291 K
0.32 × 0.28 × 0.26 mm

#### Data collection

Bruker SMART CCD diffractometer
Absorption correction: multi-scan (*SADABS*; Sheldrick, 2000 ▶) *T*_min_ = 0.936, *T*_max_ = 0.947
5345 measured reflections
3201 independent reflections
2379 reflections with *I* > 2σ(*I*)
*R*_int_ = 0.027

#### Refinement

*R*[*F* ^2^ > 2σ(*F* ^2^)] = 0.106
*wR*(*F* ^2^) = 0.198
*S* = 1.08
3201 reflections
235 parameters
H-atom parameters constrained
Δρ_max_ = 0.73 e Å^−3^
Δρ_min_ = −0.26 e Å^−3^

### 

Data collection: *SMART* (Bruker, 2000 ▶); cell refinement: *SAINT* (Bruker, 2000 ▶); data reduction: *SAINT*; program(s) used to solve structure: *SHELXTL* (Sheldrick, 2008 ▶); program(s) used to refine structure: *SHELXTL*; molecular graphics: *SHELXTL*; software used to prepare material for publication: *SHELXTL*.

## Supplementary Material

Crystal structure: contains datablocks I, global. DOI: 10.1107/S1600536810017095/ya2121sup1.cif

Structure factors: contains datablocks I. DOI: 10.1107/S1600536810017095/ya2121Isup2.hkl

Additional supplementary materials:  crystallographic information; 3D view; checkCIF report

## Figures and Tables

**Fig. 1.**
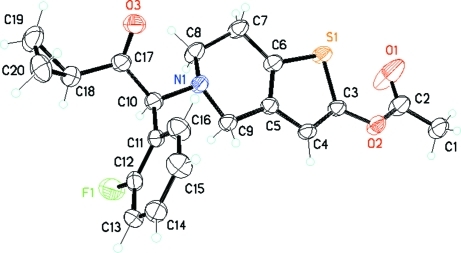
Molecular structure of the title compound with thermal ellipsoids drawn at the 30% probability level.

## supplementary crystallographic information

### Comment

Prasugrel was recently approved for clinical use in combination with aspirin as an option for preventing blockages in the arteries in patients with acute coronary syndromes who are undergoing treatment via percutaneous coronary intervention (Farid *et al.*, 2008). Both enantiomers of prasugrel show similar activity, therefore it was approved for use in its racemic form. The synthesis of prasugrel has been published recently (Sun *et al.*, 2009). Herein we report its crystal structure (Fig. 1).

The tetrahydropyridine ring of the bicyclic thienopyridine system shows a half-chair conformation with the N1 and C8 atoms displaced by -0.408 (7) Å and 0.411 (7) Å from the plane of C5, C6, C7 and C9 atoms, which are coplanar within 0.003 Å. The dihedral angle formed by the the planes of the benzene and thiophene rings (C11-C16 and C3, C4, C5, C6, S1, respectively) is equal to 83.17 (3)°.

### Experimental

The description of the seven-step synthesis of the title compound is published by Sun *et al.* (2009). Here we report the details for the two final steps of the synthesis.

Synthesis of 5-(2-cyclopropyl-1-(2-fluorophenyl)-2-oxoethyl) -5,6,7,7a-tetrahydrothieno[3,2-*c*]pyridin-2(4*H*)-one. Under N_2_ atmosphere, 5,6,7,7a-tetrahydrothieno[3,2-*c*] pyridin-2(4*H*)-one hydrochloride (19.1 g, 0.1 mol) and *N*,*N*-diisopropylformamide (27.1 g, 0.21 mol) were dissolved in 60 ml of CH_3_CN. 2-Bromo-1-cyclopropyl-2-(2-fluorophenyl)ethanone (28.1 g, 0.11 mol) was added to the solution at 40°C. The mixture was stirred for 8 h and poured into H_2_O (500 ml), then extracted with ethyl acetate (50 ml x 3). The organic phase was collected and washed with saturated NaCl solution (80 ml x 4), then dried with anhydrous Na_2_SO_4_ and filtered. The filtrate was distilled in vacuo and the solvent was removed. The residue was separated with column chromatography and pale-yellow oil of 5-(2-cyclopropyl-1-(2-fluorophenyl)-2-oxoethyl)-5,6,7,7a-tetrahydrothieno[3,\ 2-*c*]pyridin-2(4*H*)-one was obtained (12 g. 41%).

Synthesis of prasugrel. 5-(2-Cyclopropyl-1-(2-fluorophenyl)-2-oxoethyl)-5,6,7,7a-tetrahydrothieno[3,2-\ *c*]pyridin-2(4*H*)-one (3.31 g, 0.01 mol) was dissolved in the mixture of DMF (20 ml) and acetate anhydride (1.13 ml, 0.012 mol). NaH (0.44 g, 0.011 mol) was added at 0°C and stirred for 1 h at room temperature. The reaction solution was poured into iced water (50 ml) and extracted with ethyl acetate (30 ml x 3). The organic phase was separated and washed with saturated NaCl solution (50 ml x 4), then dried with anhydrous Na_2_SO_4_ and filtered. The filtrate was distilled in vacuo and the solvent was removed. The residue was washed in 10 ml of ether, and thus prasugrel, in the form of colorless solid, was obtained (2.5 g, 66%).

0.074 g (2 mmol) of prasugrel powder were dissolved in 20 ml of methanol and then slowly evaporated. After two weeks, colorless block crystals were obtained and collected [yield 83.8% (0.062 g)].

### Refinement

All the H atoms were positioned geometrically and included in the refinement using riding model approximation with C—H = 0.93–0.97 Å and  *U*_iso_(H) = 1.2*U*_eq_(C) [*U*_iso_(H) = 1.5*U*_eq_(C) for methyl H atoms]. Unfortunately, all crystals, finally formed after the prolonged crystallization, were of limited quality, which is reflected in rather poor accuracy of the structure.

### Crystal data

**Table:**

C_20_H_20_FNO_3_S	*Z* = 2
*M**_r_* = 373.43	*F*(000) = 392
Triclinic, *P*1	*D*_x_ = 1.378 Mg m^−^^3^
Hall symbol: -P 1	Mo *K*α radiation, λ = 0.71073 Å
*a* = 7.910 (2) Å	Cell parameters from 1069 reflections
*b* = 9.943 (3) Å	θ = 2.2–21.9°
*c* = 12.450 (4) Å	µ = 0.21 mm^−^^1^
α = 112.938 (5)°	*T* = 291 K
β = 90.644 (5)°	Block, colorless
γ = 92.591 (6)°	0.32 × 0.28 × 0.26 mm
*V* = 900.3 (5) Å^3^	

### Data collection

**Table:**

Bruker SMART CCD diffractometer	3201 independent reflections
Radiation source: fine-focus sealed tube	2379 reflections with *I* > 2σ(*I*)
graphite	*R*_int_ = 0.027
φ and ω scans	θ_max_ = 25.2°, θ_min_ = 1.8°
Absorption correction: multi-scan (*SADABS*; Sheldrick, 2000)	*h* = −9→9
*T*_min_ = 0.936, *T*_max_ = 0.947	*k* = −11→10
5345 measured reflections	*l* = −13→14

### Refinement

**Table:**

Refinement on *F*^2^	Primary atom site location: structure-invariant direct methods
Least-squares matrix: full	Secondary atom site location: difference Fourier map
*R*[*F*^2^ > 2σ(*F*^2^)] = 0.106	Hydrogen site location: inferred from neighbouring sites
*wR*(*F*^2^) = 0.198	H-atom parameters constrained
*S* = 1.08	*w* = 1/[σ^2^(*F*_o_^2^) + (0.0117*P*)^2^ + 3.2142*P*] where *P* = (*F*_o_^2^ + 2*F*_c_^2^)/3
3201 reflections	(Δ/σ)_max_ = 0.001
235 parameters	Δρ_max_ = 0.73 e Å^−^^3^
0 restraints	Δρ_min_ = −0.26 e Å^−^^3^

### Special details

**Table:**

Geometry. All esds (except the esd in the dihedral angle between two l.s. planes) are estimated using the full covariance matrix. The cell esds are taken into account individually in the estimation of esds in distances, angles and torsion angles; correlations between esds in cell parameters are only used when they are defined by crystal symmetry. An approximate (isotropic) treatment of cell esds is used for estimating esds involving l.s. planes.
Refinement. Refinement of *F*^2^ against ALL reflections. The weighted *R*-factor wR and goodness of fit *S* are based on *F*^2^, conventional *R*-factors *R* are based on *F*, with *F* set to zero for negative *F*^2^. The threshold expression of *F*^2^ > σ(*F*^2^) is used only for calculating *R*-factors(gt) etc. and is not relevant to the choice of reflections for refinement. *R*-factors based on *F*^2^ are statistically about twice as large as those based on *F*, and *R*- factors based on ALL data will be even larger.

### Fractional atomic coordinates and isotropic or equivalent isotropic displacement parameters (Å^2^)

**Table:**

	*x*	*y*	*z*	*U*_iso_*/*U*_eq_	
S1	0.98277 (19)	0.7094 (2)	0.12388 (13)	0.0633 (5)	
F1	0.8924 (5)	0.9710 (5)	−0.3950 (4)	0.0885 (12)	
C1	1.4971 (8)	0.5727 (9)	0.2034 (6)	0.080 (2)	
H1A	1.5091	0.5806	0.2825	0.120*	
H1B	1.5907	0.6251	0.1861	0.120*	
H1C	1.4953	0.4716	0.1514	0.120*	
C2	1.3386 (8)	0.6352 (8)	0.1886 (5)	0.074 (2)	
C3	1.1688 (7)	0.6812 (7)	0.0487 (5)	0.0526 (15)	
C4	1.1564 (7)	0.7042 (6)	−0.0503 (5)	0.0533 (15)	
H4A	1.2443	0.6929	−0.1016	0.064*	
C5	0.9935 (7)	0.7477 (6)	−0.0677 (4)	0.0482 (13)	
C6	0.8868 (7)	0.7542 (7)	0.0175 (4)	0.0528 (15)	
C7	0.7093 (7)	0.8012 (8)	0.0226 (5)	0.0643 (18)	
H7A	0.6854	0.8667	0.1014	0.077*	
H7B	0.6301	0.7167	−0.0004	0.077*	
C8	0.6913 (7)	0.8786 (7)	−0.0600 (5)	0.0625 (17)	
H8A	0.5732	0.8971	−0.0675	0.075*	
H8B	0.7554	0.9719	−0.0289	0.075*	
C9	0.9400 (7)	0.7866 (7)	−0.1670 (5)	0.0538 (15)	
H9A	0.9894	0.8825	−0.1555	0.065*	
H9B	0.9814	0.7164	−0.2392	0.065*	
C10	0.7071 (7)	0.8485 (6)	−0.2608 (5)	0.0491 (14)	
H10A	0.7532	0.9498	−0.2343	0.059*	
C11	0.7730 (6)	0.7596 (6)	−0.3819 (4)	0.0437 (13)	
C12	0.8627 (7)	0.8249 (6)	−0.4420 (5)	0.0475 (13)	
C13	0.9202 (7)	0.7512 (7)	−0.5530 (5)	0.0574 (16)	
H13A	0.9813	0.8007	−0.5910	0.069*	
C14	0.8856 (8)	0.6067 (8)	−0.6044 (5)	0.0620 (16)	
H14A	0.9245	0.5550	−0.6787	0.074*	
C15	0.7937 (9)	0.5342 (8)	−0.5491 (6)	0.0714 (19)	
H15A	0.7685	0.4341	−0.5863	0.086*	
C16	0.7382 (9)	0.6108 (7)	−0.4370 (5)	0.0679 (18)	
H16A	0.6771	0.5613	−0.3990	0.082*	
C17	0.5147 (7)	0.8444 (7)	−0.2809 (5)	0.0517 (14)	
C18	0.4630 (8)	0.9356 (7)	−0.3427 (5)	0.0654 (17)	
H18A	0.5474	1.0094	−0.3454	0.079*	
C19	0.2829 (9)	0.9711 (9)	−0.3429 (6)	0.089 (2)	
H19A	0.2054	0.9357	−0.2987	0.106*	
H19B	0.2590	1.0659	−0.3429	0.106*	
C20	0.3480 (9)	0.8650 (9)	−0.4476 (6)	0.085 (2)	
H20A	0.3651	0.8935	−0.5130	0.102*	
H20B	0.3115	0.7632	−0.4688	0.102*	
N1	0.7541 (5)	0.7875 (5)	−0.1760 (4)	0.0494 (12)	
O1	1.2404 (8)	0.6812 (10)	0.2594 (4)	0.168 (4)	
O2	1.3121 (5)	0.6278 (5)	0.0798 (3)	0.0711 (13)	
O3	0.4151 (5)	0.7686 (5)	−0.2536 (4)	0.0692 (12)	

### Atomic displacement parameters (Å^2^)

**Table:**

	*U*^11^	*U*^22^	*U*^33^	*U*^12^	*U*^13^	*U*^23^
S1	0.0504 (9)	0.1083 (14)	0.0387 (8)	0.0199 (9)	0.0111 (6)	0.0349 (8)
F1	0.092 (3)	0.092 (3)	0.090 (3)	0.005 (2)	0.024 (2)	0.043 (3)
C1	0.070 (4)	0.121 (6)	0.063 (4)	0.032 (4)	0.006 (3)	0.047 (4)
C2	0.064 (4)	0.112 (6)	0.045 (4)	0.027 (4)	0.005 (3)	0.027 (4)
C3	0.045 (3)	0.078 (4)	0.039 (3)	0.014 (3)	0.009 (2)	0.027 (3)
C4	0.047 (3)	0.075 (4)	0.044 (3)	0.016 (3)	0.014 (3)	0.027 (3)
C5	0.046 (3)	0.061 (4)	0.039 (3)	0.009 (3)	0.004 (2)	0.021 (3)
C6	0.047 (3)	0.076 (4)	0.034 (3)	0.009 (3)	0.002 (2)	0.019 (3)
C7	0.047 (3)	0.106 (5)	0.040 (3)	0.022 (3)	0.008 (3)	0.027 (3)
C8	0.053 (4)	0.085 (5)	0.040 (3)	0.024 (3)	0.002 (3)	0.012 (3)
C9	0.048 (3)	0.077 (4)	0.042 (3)	0.014 (3)	0.009 (3)	0.028 (3)
C10	0.048 (3)	0.057 (4)	0.044 (3)	0.003 (3)	0.001 (2)	0.022 (3)
C11	0.038 (3)	0.058 (4)	0.041 (3)	0.004 (2)	−0.001 (2)	0.026 (3)
C12	0.048 (3)	0.045 (3)	0.055 (3)	0.004 (3)	−0.003 (3)	0.026 (3)
C13	0.054 (4)	0.080 (5)	0.053 (4)	0.012 (3)	0.012 (3)	0.042 (4)
C14	0.066 (4)	0.075 (5)	0.047 (3)	0.014 (3)	0.007 (3)	0.024 (3)
C15	0.087 (5)	0.065 (4)	0.057 (4)	−0.001 (4)	0.004 (4)	0.018 (3)
C16	0.084 (5)	0.074 (5)	0.047 (4)	0.005 (4)	0.007 (3)	0.024 (3)
C17	0.048 (3)	0.069 (4)	0.038 (3)	0.005 (3)	0.003 (3)	0.021 (3)
C18	0.052 (4)	0.086 (5)	0.068 (4)	0.005 (3)	−0.002 (3)	0.041 (4)
C19	0.066 (5)	0.135 (7)	0.072 (5)	0.027 (5)	−0.006 (4)	0.047 (5)
C20	0.100 (6)	0.108 (6)	0.057 (4)	0.007 (5)	−0.014 (4)	0.043 (4)
N1	0.045 (3)	0.069 (3)	0.037 (2)	0.015 (2)	0.005 (2)	0.022 (2)
O1	0.121 (5)	0.333 (10)	0.051 (3)	0.131 (6)	0.019 (3)	0.063 (5)
O2	0.060 (3)	0.117 (4)	0.050 (2)	0.039 (3)	0.012 (2)	0.044 (3)
O3	0.049 (2)	0.111 (4)	0.061 (3)	−0.007 (2)	−0.005 (2)	0.051 (3)

### Geometric parameters (Å, °)

**Table:**

S1—C3	1.727 (5)	C10—N1	1.460 (7)
S1—C6	1.731 (5)	C10—C11	1.531 (7)
F1—C12	1.346 (6)	C10—C17	1.535 (7)
C1—C2	1.464 (8)	C10—H10A	0.9800
C1—H1A	0.9600	C11—C12	1.355 (7)
C1—H1B	0.9600	C11—C16	1.380 (8)
C1—H1C	0.9600	C12—C13	1.381 (8)
C2—O1	1.150 (7)	C13—C14	1.338 (8)
C2—O2	1.342 (7)	C13—H13A	0.9300
C3—C4	1.342 (7)	C14—C15	1.368 (9)
C3—O2	1.385 (6)	C14—H14A	0.9300
C4—C5	1.418 (7)	C15—C16	1.392 (8)
C4—H4A	0.9300	C15—H15A	0.9300
C5—C6	1.346 (7)	C16—H16A	0.9300
C5—C9	1.494 (7)	C17—O3	1.206 (7)
C6—C7	1.494 (7)	C17—C18	1.467 (8)
C7—C8	1.515 (8)	C18—C19	1.483 (8)
C7—H7A	0.9700	C18—C20	1.492 (9)
C7—H7B	0.9700	C18—H18A	0.9800
C8—N1	1.480 (6)	C19—C20	1.438 (9)
C8—H8A	0.9700	C19—H19A	0.9700
C8—H8B	0.9700	C19—H19B	0.9700
C9—N1	1.474 (6)	C20—H20A	0.9700
C9—H9A	0.9700	C20—H20B	0.9700
C9—H9B	0.9700		
			
C3—S1—C6	90.2 (3)	C17—C10—H10A	109.4
C2—C1—H1A	109.5	C12—C11—C16	116.7 (5)
C2—C1—H1B	109.5	C12—C11—C10	121.3 (5)
H1A—C1—H1B	109.5	C16—C11—C10	121.9 (5)
C2—C1—H1C	109.5	F1—C12—C11	119.3 (5)
H1A—C1—H1C	109.5	F1—C12—C13	116.8 (5)
H1B—C1—H1C	109.5	C11—C12—C13	123.9 (6)
O1—C2—O2	121.5 (6)	C14—C13—C12	118.3 (6)
O1—C2—C1	125.3 (6)	C14—C13—H13A	120.9
O2—C2—C1	113.1 (5)	C12—C13—H13A	120.9
C4—C3—O2	122.4 (5)	C13—C14—C15	120.9 (6)
C4—C3—S1	112.7 (4)	C13—C14—H14A	119.6
O2—C3—S1	124.6 (4)	C15—C14—H14A	119.6
C3—C4—C5	112.1 (5)	C14—C15—C16	119.7 (7)
C3—C4—H4A	123.9	C14—C15—H15A	120.1
C5—C4—H4A	123.9	C16—C15—H15A	120.1
C6—C5—C4	113.0 (5)	C11—C16—C15	120.5 (6)
C6—C5—C9	121.4 (5)	C11—C16—H16A	119.7
C4—C5—C9	125.6 (5)	C15—C16—H16A	119.7
C5—C6—C7	124.1 (5)	O3—C17—C18	122.7 (5)
C5—C6—S1	112.0 (4)	O3—C17—C10	123.7 (5)
C7—C6—S1	123.9 (4)	C18—C17—C10	113.6 (5)
C6—C7—C8	108.0 (5)	C17—C18—C19	119.3 (6)
C6—C7—H7A	110.1	C17—C18—C20	117.3 (6)
C8—C7—H7A	110.1	C19—C18—C20	57.8 (4)
C6—C7—H7B	110.1	C17—C18—H18A	116.5
C8—C7—H7B	110.1	C19—C18—H18A	116.5
H7A—C7—H7B	108.4	C20—C18—H18A	116.5
N1—C8—C7	110.0 (5)	C20—C19—C18	61.4 (5)
N1—C8—H8A	109.7	C20—C19—H19A	117.6
C7—C8—H8A	109.7	C18—C19—H19A	117.6
N1—C8—H8B	109.7	C20—C19—H19B	117.6
C7—C8—H8B	109.7	C18—C19—H19B	117.6
H8A—C8—H8B	108.2	H19A—C19—H19B	114.7
N1—C9—C5	111.1 (4)	C19—C20—C18	60.8 (4)
N1—C9—H9A	109.4	C19—C20—H20A	117.7
C5—C9—H9A	109.4	C18—C20—H20A	117.7
N1—C9—H9B	109.4	C19—C20—H20B	117.7
C5—C9—H9B	109.4	C18—C20—H20B	117.7
H9A—C9—H9B	108.0	H20A—C20—H20B	114.8
N1—C10—C11	111.6 (4)	C10—N1—C9	109.7 (4)
N1—C10—C17	112.8 (5)	C10—N1—C8	109.8 (4)
C11—C10—C17	104.1 (4)	C9—N1—C8	108.6 (4)
N1—C10—H10A	109.4	C2—O2—C3	121.6 (4)
C11—C10—H10A	109.4		
